# The prognostic value of ADAMTS13 (a disintegrin and metalloprotease with thrombospondin type 1 repeats, member 13) deficiency in septic shock patients involves interleukin-6 and is not dependent on disseminated intravascular coagulation

**DOI:** 10.1186/cc13115

**Published:** 2013-11-18

**Authors:** Vincent Peigne, Elie Azoulay, Isaline Coquet, Eric Mariotte, Michael Darmon, Paulette Legendre, Nadir Adoui, Anne Marfaing-Koka, Martine Wolf, Benoit Schlemmer, Agnès Veyradier

**Affiliations:** 1 Inserm U770, Le Kremlin Bicêtre, Paris, France; 2 Service de Réanimation Médicale, Hôpital Saint Louis, AP-HP, Paris et Faculté de Médecine Paris 7, Paris, France; 3 Service de Biochimie, Hôpital Saint Louis, AP-HP, Paris et Faculté de Médecine Paris 7, Paris, France; 4 Service d’Hématologie biologique, Hôpital Antoine Béclère, AP-HP, Clamart et Faculté de Médecine Paris 11, Le Kremlin Bicêtre, France; 5 AP-HP, Hôpital Saint-Louis, Medical ICU, Université Paris-Diderot, Sorbonne Paris-Cité, Faculté de Médecine, 1 avenue Claude Vellefaux, 75010, Paris, France

## Abstract

**Introduction:**

ADAMTS13 (a disintegrin and metalloprotease with thrombospondin type 1 repeats, member 13) deficiency has been reported in patients with sepsis but its clinical relevance and pathophysiology remain unclear. Our objectives were to assess the clinical significance, prognostic value and pathophysiology of ADAMTS13 deficiency in patients with septic shock with and without disseminated intravascular coagulation (DIC).

**Methods:**

This was a prospective monocenter cohort study of patients with septic shock. Von Willebrand Factor, ADAMTS13-related parameters and plasma IL-6 concentration were measured at inclusion to the study. Patients were categorized into three groups according to the presence of ADAMT13 deficiency (<30%) or DIC.

**Results:**

This study included 72 patients with a median age of 59 years (interquartile range (IQR) 50 to 71). Each of the included patients received vasopressors; 55 (76%) were under mechanical ventilation and 22 (33%) underwent renal replacement therapy. Overall, 19 patients (26%) had DIC, and 36 patients had ADMTS13 deficiency (50%). Patients with DIC, ADAMTS13 deficiency or both were more severe at ICU admission. Mortality was higher in septic shock patients from group one. By multivariate analysis, Simplified Acute Physiology Score 2 (SAPS2) score (odds ratio (OR) 1.11/point; 95% CI 1.01 to 1.24) and ADAMTS13 activity <30% (OR 11.86; 95% CI 1.36 to 103.52) were independently associated with hospital mortality. There was no correlation between ADAMTS13 activity and the International Society for Thrombosis and Haemostasis (ISTH) score (r_s =_ -0.97, *P =* 0.41) suggesting that ADAMTS13 functional deficiency and DIC were independent parameters. IL-6 level was higher in patients with ADAMTS13 activity <30% [895 (IQR 330 to 1843) pg/mL versus 83 (IQR 43 to 118), *P =* 0.0003).

**Conclusions:**

Septic shock was associated with a functional deficiency of ADAMTS13, independently of DIC. ADAMTS13 functional deficiency is then a prognostic factor for mortality in septic shock patients, independently of DIC.

## Introduction

Sepsis is most frequently associated with hemostasis abnormalities [1-3], especially disseminated intra-vascular coagulation (DIC). DIC is characterized by the association of systemic activation of coagulation, impairment of anticoagulant pathways and inhibition of fibrinolysis [4,5]. Manifestations of DIC, which is associated with an increased mortality during sepsis, include bleeding and microthrombi leading to organ failure [6]. Animal studies and clinical trials reported that impairment of several anticoagulant pathways, like Protein C [7,8], tissue factor pathway inhibitor [9] or anti-thrombin [8], could also occur during sepsis. Substitution therapy with these factors has not been shown to improve sepsis outcome [10,11].

Beside coagulation disturbances, abnormalities of von Willebrand factor (VWF), related to endothelial damage, have been reported in sepsis patients during the last decade [12,13]. VWF is a multimeric glycoprotein crucial for platelet adhesion and aggregation at the high shear rates of blood flow present in the microcirculation. The largest VWF multimers are the most adhesive to platelets and thus, the most efficient for primary hemostasis. Ultra-large VWF multimers (ULVWF) are physiologically present in endothelial cells and platelets, but absent in plasma. A metalloprotease, ADAMTS13 (a disintegrin and metalloprotease with thrombospondin type 1 repeats, member 13), specifically cleaves ULVWF multimers and thus, regulates its adhesive function. A severe ADAMTS13 deficiency (activity <5%) either inherited or most often acquired via specific autoantibodies, is the main actor in the pathophysiology of thrombotic thrombocytopenic purpura (TTP) [14,15]. TTP is a thrombotic microangiopathy (TMA) characterized by thrombopenia, mechanical hemolytic anemia, organ failure and VWF-rich microthrombi [16-18]. As a consequence of its implication on VWF multimer synthesis, sepsis has been reported as one of the factors that might trigger thrombotic microangiopathy [19] and TTP is one of the differential diagnoses of DIC. Some cases of ADAMTS13 deficiency have been reported recently in adult patients with sepsis-induced DIC and this ADAMTS13 deficiency was associated with acute renal failure [20]. Later clinical studies, also in adults and focusing on sepsis without DIC [21-25] or isolated DIC not specifically related to sepsis [26], reported decreased ADAMTS13 activity. Interestingly, some of these studies [20,21,25,26] showed that partially decreased ADAMTS13 activity was associated with outcome. In pediatric patients with severe sepsis, ADAMTS13 deficiencies have also been reported [27-29]. In one of the latter studies focused on children with thrombocytopenia-associated multiple organ failure, mortality was associated with reduced ADAMTS13 activity and, interestingly, with VWF-rich microvascular thrombosis at autopsy [28]. Also, this study demonstrated that plasma exchange, a source of active ADAMTS13, was able to restore ADAMTS13 activity and organ function [28].

Although these studies gave interesting insight into ADAMTS13 levels during sepsis, clinical relevancy, prognostic association and mechanisms of a potential deficiency remain uncertain. We thus designed the current prospective study with the primary objective to study the interaction between ADAMTS13 and DIC during septic shock and its impact on outcome. As a secondary objective, we investigated the mechanisms for ADAMTS13 functional variations in this clinical context. DIC, VWF and ADAMTS13 parameters were exhaustively analyzed and correlated with clinical data in 72 patients with septic shock prospectively enrolled from a single Medical ICU.

## Methods

### Patients

This study was approved by the Ethics Committee of the SRLF, the French Speaking Society of Critical Care Medicine (Approval number SRLF-CE 07–188) and the need for informed consent was waived according to French Law. Every of the included patients, or patients' next-of-kin were informed however, and none refused to participate in this study. Seventy-two consecutive patients with septic shock were prospectively enrolled at the Medical ICU of Saint-Louis Hospital, Paris, France, between March 2007 and February 2008. Septic shock was diagnosed according to the American College of Chest Physicians/Society of Critical Care Medicine definitions [30]. Clinical data, including sex, age, blood cell count, Simplified Acute Physiology Score II (SAPS II) [31] and Logistic Organ Dysfunction (LOD) [32] scores, requirement for mechanical ventilation or renal replacement therapy, were analyzed. Five mL of citrated venous blood were collected at day 1 of admission within 12 hours of the diagnosis of septic shock and before medication. Platelet-poor plasma was obtained after two centrifugations at 1200 g and frozen at -80°C until testing.

### DIC diagnosis

DIC was diagnosed using the International Society for Thrombosis and Haemostasis (ISTH) DIC score [33], which has been prospectively validated in intensive care patients [34]. The score ranges from 0 to 8. A score ≥5 is compatible with overt DIC in patients with an underlying disorder known to be associated with DIC. Calculation of the score requires only routine coagulation tests: platelets count, assay of a fibrin-related marker, prothrombin time and fibrinogen. D-dimers were used as a fibrin-related marker. As the prothrombin index (PI) was available instead of prothrombin time, we used the thresholds defined by Angstwurm *et al*. [35] for scoring (that is, PI >70%: 0 points; 40 to 70%: 1 point; <40%: 2 points).

### Laboratory methods

For VWF antigen measurement, plasma VWF antigen (VWF:Ag) was quantified using the STA-Liatest VWF® (Diagnostica Stago, Asnières, France) immunoturbidimetric assay [36]. Samples were compared to a normal plasma pool and results were expressed as percentages (normal range: 50 to 150%). For VWF multimeric distribution, VWF multimer analysis was performed as previously described using standard sodium dodecylsulfate (SDS)/agarose gel electrophoresis, followed by immunoblotting [37,38]. ADAMTS13 activity was determined using the synthetic fluorescent substrate FRETS-VWF73 as previously described [39] (normal range: 50 to 150%). For ADAMTS13 antigen measurement, plasma ADAMTS13 antigen was determined using the IMUBIND ADAMTS13 ELISA® kit (American Diagnostica, Stamford, CT, USA) (normal range: 540 ± 190 ng/mL). ADAMTS13 autoantibodies were screened in all patients using the TECHNOZYME ADAMTS13-INH ELISA® commercial kit (Technoclone, Vienna, Austria) which allows titration of specific anti-ADAMTS13 immunoglobulin (Ig)G (positive if >12 U/mL, according to the manufacturer’s instructions). In both patients in whom the titer of anti-ADAMTS13 IgG was positive and/or whose ADAMTS13 activity was below the median, an inhibitor assay was performed as previously described [40]. Plasma IL-6 was determined using the Human IL-6 Quantikine ELISA kit from R&D Systems (Minneapolis, MN, USA).

### Statistical analysis

Data are reported as numbers (percentages) or medians (IQR: 25th to 75th percentiles). Continuous variables were compared using the Wilcoxon rank sum test and proportions using the Fisher exact test. Hospital mortality was the outcome variable of interest. We performed univariate logistic regression analysis to identify variables that significantly influenced the likelihood of mortality, as measured by the estimated odds ratio (OR) with the 95% CI. All variables have been tested in univariate analysis. Variables of interest were defined prior to study initiation. Logistic regression analysis was then performed to identify variables significantly associated with hospital mortality, as measured by the estimated OR with the 95% CI. Variables yielding *P*-values lower than 0.20 in the bivariate analyses were entered into backward stepwise logistic regression models where hospital mortality was the variable of interest. The critical *P*-value for removal was 0.1. Co-linearity and interactions were tested. The Hosmer-Lemeshow test was used to check goodness of fit. Given the number of events, a maximum of three variables was allowed in the models. Overall survival was estimated using the Kaplan-Meier method then compared using the log-rank test. Analyses were done using the Statview software package (SAS Institute, Carry, NC, USA).

## Results

### Baseline characteristics and clinical course

Out of 72 enrolled patients, 46 (64%) were men. Median (IQR) age was 59 (50 to 71) years. Bacterial infection was documented in 44 (61%) patients. SAPS II and LOD scores at admission were 56 (45 to 71) and 7 (5 to 10), respectively. Among the 72 patients, 46 had microbiological documentation of their septic shock, including, 22 with pneumonia, 11 with pyelonephritis, 7 with colitis and 6 with catheter-related infections. Pathogens involved were *Escherichia coli* (n = 12), *Streptococcus pneumoniae* (n = 11), *Pseudomonas aeruginosa* (n = 7), Streptococcus species (n = 5), *Staphylococcus aureus* (n = 4) and miscellaneous (n =7), namely, *Salmonella typhi* (n = 2), *Neisseria meningitides* (n = 2), *Klebsiella pneumonia* (n = 2) and *Acinetobacter baumannii* (n = 1).

The ISTH score was >5 in 19 (26%) patients who therefore had overt DIC. Among detectable values (in 68 patients out of 72), median ADAMTS13 activity was 30% (IQR 19 to 45, range 10 to 78) whereas ADAMTS13 activity was undetectable (<5%) in 4 patients out of 72. Among these four latter patients, none had DIC and VWF/ADAMTS13 parameters were not significantly different from the rest of the cohort; all of them required mechanical ventilation and three of them died. In the whole cohort, median ADAMTS13:Ag level was 487 ng/mL (IQR 406 to 654, range 216 to 1965) and median VWF:Ag level was 516% (IQR 371 to 695, range 234 to 1194).

All patients received vasopressors, 55 (76%) were mechanically ventilated, and 22 (33%) underwent renal replacement therapy. ICU and hospital mortality rates were 40.3% (29 deaths) and 54.2% (39 deaths), respectively.

### Respective influence of DIC and ADAMTS13 on clinical presentation and prognosis

There was no correlation between ADAMTS13 activity and the ISTH score (*r*_s_ -0.97, *P =* 0.41), suggesting that ADAMTS13 functional deficiency and DIC were independent parameters. Comparison between patients with DIC (n = 19) and those without DIC (n = 53) showed no significant difference, especially in ADAMTS13 and VWF. In contrast, comparison between patients with an ADAMTS13 activity <30% (n = 36) and those whose ADAMTS13 activity was > 30% (n = 36) exhibited a significant difference for both persistent shock at day 3 (88% versus 41%, *P* = 0.0006) and mortality (74% versus 41%, *P* = 0.03) (Table 1).

**Table 1:** Comparison of biological parameters in patients as a function of the 30% median threshold of ADAMTS13 activity

**Median (IQR) or number (%)**	**Patients with ADAMTS13 activity <30% (n = 36)**	**Patients with ADAMTS13 activity >30% (n = 36)**	***P*-value**
DIC	11 (31%)	8 (22%)	0.59
ISTH score	3 (2, 5)	3 (1, 4)	0.58
Platelet count, day 1	61 (27, 199)	136 (29, 191)	0.47
Platelet count <65 G/L	17 (47.2%)	20 (56%)	0.05
ADAMTS13:Ag, ng/mL	457 (374, 496)	533 (413, 680)	0.15
Positive anti-ADAMTS13 IgG	3	4	NA
Anti-ADAMTS13 inhibitor	0	0	NA
VWF:Ag, %	492 (369, 642)	560 (402, 700)	0.42
IL-6, pg/mL	895 (330, 1843)	83 (43, 118)	0.0003

Values are expressed as median and IQR for continuous variables and as number and proportion for categorical variables. Continuous variables were compared using the non-parametric Mann–Whitney *U*-test. Categorical variables were compared using the *χ*^2^ test. *P* <0.05 was considered significant. DIC, disseminated intravascular coagulation; ISTH: International Society for Thrombosis and Haemostasis; ADAMTS13, a disintegrin and metalloprotease with thrombospondin type 1 repeats, member 13; Ig, immunoglobulin; VWF:Ag, von Willebrand Factor antigen; NA, statistical test not performed.

### Impact of the coupled ADAMTS13 activity/DIC on prognosis

To respond to our primary objective focused on both the interaction between DIC and ADAMTS13 and prognosis, we decided to divide the cohort into three groups. As shown in Table 2, among the 72 patients with septic shock, 28 (39%) patients had no DIC and an ADAMTS activity above the median (group 1); 33 (46%) patients had either DIC (n = 8) or an ADAMTS13 activity below the median (n = 25) (group 2), and 11 (15%) patients had both DIC and an ADAMTS13 activity below the median (group 3). Four patients had ADAMTS13 activity <5%.

**Table 2:** Severity of disease at presentation and outcome according to the number of hemostatic abnormalities in 72 patients with septic shock

**Median (IQR) or number (%)**	**Group 1, n = 28 (no DIC or ADAMTS13 activity >30%**^**a**^**)**	**Group 2, n = 33 (either DIC or ADAMTS13 activity <30%**^**a**^**)**	**Group 3, n = 11 (both DIC and ADAMTS13 activity <30%**^**a**^**)**	***P*-value**
ISTH score	3 (2, 4)	3 (2, 5)	5 (5, 7)	0.01
ADAMTS13 activity	46% (39, 79)	24% (19, 54)	15% (9, 25)	0.002
**Severity of disease**				
LOD score, day 1	7 (4, 9)	7 (6, 10)	13 (7, 14)	0.02
SAPS II score, day 1	48 (38, 65)	62 (47, 69)	77 (51, 95)	0.01
Systolic blood pressure, mmHg	85 (80, 91)	80 (67, 86)	72 (67, 76)	0.004
PaO/FiO2	240 (116, 300)	140 (110, 245)	95 (76, 127)	0.02
Lactates, mmol/L	2.6 (2.0, 5.6)	3 (1.9, 6.3)	6.4 (1.9, 16.5)	0.06
IL-6 concentration, pg/mL	78 (46, 126)	318 (94, 1003)	1,835 (500, 3,030)	0.004
**Outcome**				
Need for mechanical ventilation	19 (68)	26 (79)	10 (91)	0.25
Persistent shock, day 3	10 (35.7)	26 (79)	11 (100)	0.001
Need for RRT	8 (28.6)	10 (30.3)	5 (45.3)	0.24
Continuous hemofiltration	2 (7.1)	6 (18.2)	4 (36.3)	0.09
Dotrecogrin alpha activated	2 (7.1)	0	0	0.19
Stress dose steroids	6 (21.4)	6 (18.2)	1 (9)	0.64
Length of ICU stay	5 (3, 13)	5 (3, 13)	6 (2, 15)	0.95
ICU mortality	6 (21.4)	18 (54.5)	5 (45.4)	0.02
Hospital mortality	28.6%	71%	75%	0.007

Values are expressed as median and IQR for continuous variables and as proportion for categorical variables. Continuous variables were compared using the non-parametric Kruskall-Wallis test. Categorical variables were compared using thea *χ*^2^ test. *P* <0.05 was considered significant. The normal range for ADAMTS13 activity is 50 to 150%. ^a^30% corresponds to the median of ADAMTS13 activity in the 72 patients with septic shock. DIC, disseminated intravascular coagulation; ISTH: International Society for Thrombosis and Haemostasis; ADAMTS13, a disintegrin and metalloprotease with thrombospondin type 1 repeats, member 13; LOD, logistic organ dysfunction; SAPS II, Simplified Acute Physiology Score II; PaO/FiO2, arterial partial pressure of oxygen/inspired oxygen fraction; RRT, renal replacement therapy.

Patients with both DIC and ADAMTS13 activity <30% had higher SAPS II and LOD severity scores than other patients (77 versus 62 and 48, respectively for SAPS II, *P =* 0.01 and 13 versus 7 and 7, respectively *P =* 0.01 for LOD). The more hemostatic abnormalities the patients had, the lower the blood pressure was (group 1, 85 mmHg; group 2, 80 mmHg; group 3, 72 mmHg) and the arterial partial pressure of oxygen/inspired oxygen fraction (PaO_2_/FiO_2_) ratio (group 1, 240; group 2, 140; group 3, 95). There was a trend toward higher lactate level in group-3 patients (6.4 mmol/L versus 3.0 in group-1 and 2.4 in group-2 patients, *P =* 0.06). Median IL-6 concentration significantly increased across the groups (group 1, 78 pg/mL; group 2, 318 pg/mL; group 3, 1835 pg/mL, *P =* 0.004).

#### ***Outcomes***

The need for mechanical ventilation and dialysis did not differ among the three groups. However group-3 patients without prior chronic renal failure required dialysis more frequently than other patients without prior chronic renal failure (71.4%, 50% and 50%, respectively, *P* = 0.05). Persistent shock at day 3 was more frequent in group-3 and group-2 patients than in group-1 patients (100%, 78% and 38%, respectively, *P* <0.001). Group-3 patients were more frequently hypothermic and had more frequent positive blood cultures.

#### ***Influence of ADAMTS13 on outcome***

**Figure 1:**
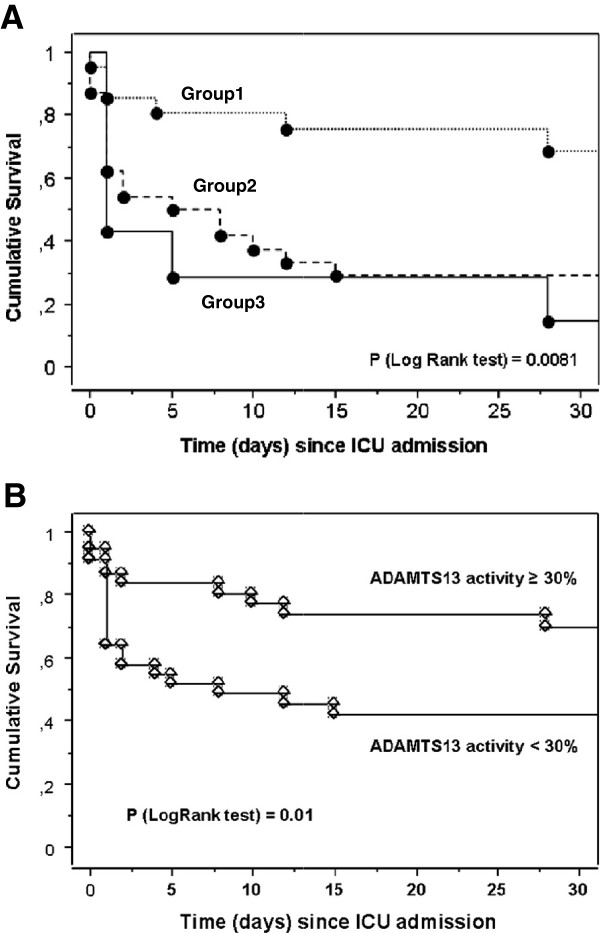
**Kaplan-Meier graph for cumulative survival according to the number of hemostatic abnormalities (A) or according to ADAMTS13 activity (B) in 72 patients with septic shock.** Group 1: no DIC and ADAMTS13 activity >30%; group 2: either DIC or ADAMTS13 activity <30%; group 3: DIC and ADAMTS13 activity <30%: 30% corresponds to the median of ADAMTS13 activity in the 72 patients with septic shock. ADAMTS13, a disintegrin and metalloprotease with thrombospondin type 1 repeats, member 13; DIC, disseminated intravascular coagulation.

ICU mortality and hospital mortality were significantly higher in group-2 and -3 patients than in group-1 patients (Table 2, Figure 1).

A comparison between ICU survivors (n = 43) and non-survivors (n = 29) is presented in Table 3. Univariate analysis of biological parameters showed that mortality was associated with ADAMTS13 activity <30%, higher VWF:Ag level and higher IL-6 level. Results of the logistic regression are reported in Table 4. Both SAPS II score and ADAMTS13 activity lower than 30% were independently associated with hospital mortality.

**Table 3:** Comparison of biological parameters between survivors and non survivors (ICU mortality) of the septic shock cohort (n = 72)

	**Survivors**	**Non-survivors**	***P*-value**
	**(n = 43)**	**(n = 29)**	
**Disseminated intravascular coagulation (DIC)**	10/43 (23%)	9/29 (31%)	0.59
**ISTH score**	3 (1, 4)	4 (2, 5)	0.67
**Platelet count, G/l**	65 (20, 184)	87 (47, 195)	0.30
**ADAMTS13 activity <30%**	17/43 (37%)	19/29 (66%)	0.05
**ADAMTS13 activity <5%**	1/43	3/29	-
**ADAMTS13:Ag, ng/mL**	483 (384, 613)	491 (418, 696)	0.24
**VWF:Ag, %**	477 (341, 656)	560 (478, 835)	0.05
**IL-6, pg/mL**	107 (48, 418)	840 (204, 1089)	0.03

Values are expressed as median and interquartile range for continuous variables and as number and proportion for categorical variables. Continuous variables were compared using the non-parametric Mann–Whitney *U*-test. Categorical variables were compared using the *χ*^2^ test. *P* <0.05 was considered significant. ISTH, International Society for Thrombosis and Haemostasis; ADAMTS13, a disintegrin and metalloprotease with thrombospondin type 1 repeats, member 13; ADAMTS13:Ag, ADAMTS13 antigen; VWF:Ag, von Willebrand Factor antigen.

**Table 4:** Factors independently associated with hospital mortality (conditional backward stepwise regression model)

	**Odds ratio**	**95% CI**	**P-value**
**SAPS II score, per point**	1.11	1.01, 1.24	<0.0001
**ADAMTS13 activity <30%**	11.86	1.36-103-52	0.0004

SAPS II, Simplified Acute Physiology Score II; ADAMTS13, a disintegrin and metalloprotease with thrombospondin type 1 repeats, member 13.

Performance of ADAMTS13 activity for the prediction of hospital mortality has also been assessed using receiver operating characteristic (ROC) curves. The area under the curve was 0.74 (0.60 to 0.88), with 67% sensitivity, 69% specificity, a Younden index of 0.36, a positive predictive value of 0.67 and a negative predictive value of 0.70.

### Mechanisms for ADAMTS13 deficiency

#### ***Consumption of ADAMTS13 by high levels of its substrate, VWF***

Plasma VWF:Ag was not significantly different across the three groups (group 1, 578%, group 2, 543%, group 3, 393%, *P* = 0.14) as well as between patients with ADAMTS13 activity <30% and those with ADAMTS13 activity >30% (Table 1). Whatever the patient group, the VWF multimeric pattern was similar, systematically showing the presence of ULVWF.

#### ***Synthesis/secretion defect of ADAMTS13, proteolytic degradation of ADAMTS13***

Overall, ADAMTS13:Ag was not correlated with ADAMTS13 activity. Also, it was not significantly different as a function of ADAMTS13 activity towards the 30% median (Table 1) or across the three groups.

#### ***Functional inactivation of ADAMTS13***

Detection of anti-ADAMTS13 IgG was positive in seven patients: three exhibited ADAMTS13 activity <30% (15%, 24%, and 26%, respectively) and four exhibited ADAMTS13 activity >30%. In all of them, no circulating inhibitor could be detected. Similarly, the inhibitor assay was negative in all patients whose ADAMTS13 activity was <30%. IL-6 concentration was significantly different between the three groups (group 1, 78 pg/mL; group 2, 318 pg/mL; group 3, 1835 pg/ml, *P* = 0.004) (Table 2) and clearly increased from group 1 to 3. Interestingly, IL-6 level was considerably higher in patients whose ADAMTS13 activity was <30% than in patients with ADAMTS13 activity >30% (median 895 (IQR 330 to 1843) pg/mL and 83 (IQR 43 to 118), *P* = 0.0003) (Table 1).

## Discussion

Sepsis-induced organ failure is associated with a microcirculation damage process involving mainly platelet/fibrin-thrombi supporting DIC. In some cases, the potential addition of platelet/VWF thrombi due to an acquired ADAMTS13 functional deficiency and leading to so-called TMA-like lesions is highly suspected in clinical arguments [41-43] but still remains debated in terms of prognosis and pathophysiology. Furthermore, in most studies published so far, the subtle combination of DIC and TMA-like lesions has been difficult to establish because of the great heterogeneity of the sepsis cohorts tested, involving mixed cases within the categories of systemic inflammation, sepsis, septic shock, and sepsis-related multiple organ failure [20-26], some of them complicated by DIC and others not. In the current study, we focused on a cohort of 72 patients with septic shock prospectively enrolled from a single ICU in order to respond to two objectives: first, to analyze the interaction between DIC and ADAMTS13 and its impact on prognosis, and second, to elucidate the pathophysiological mechanisms for ADAMTS13 deficiency. The strength of our study is to propose an approach based on clinical practice supported by three groups to evaluate the prognostic value of ADAMTS13 in sepsis: we showed that septic shock outcome is associated with a functional deficiency of ADAMTS13 together with an increase of IL-6, independently of DIC.

In our cohort, the median of ADAMTS13 activity was 30%, which is in agreement with the literature [20-26] and most patients (57/72 = 79%) exhibited partial ADAMTS13 functional deficiency (values <50%). DIC was present in 19 patients (26%). The overlap between DIC and ADAMTS13 activity <30% was present only in 11 patients (15%), suggesting that these biological parameters were not linked, which was further confirmed by the absence of correlation between the ISTH score and ADAMTS13 activity and by the lack of a significant difference in ADAMTS13 between DIC-positive and DIC-negative patients and in the ISTH score between patients with ADAMTS13 activity >30% and <30%. Thus, the independence between DIC and ADAMTS13 activity prompted us to divide our cohort into three groups in order to compare patients with absence of both DIC and ADAMTS13 deficiency to other patients. We observed that the higher the number of hemostatic abnormalities, the higher the disease severity. We also found a strong significant difference between groups for all indicators of disease severity at ICU admission (LOD score, SAPS II score, systolic blood pressure, PaO/FiO2, lactates, IL-6 concentrations) suggesting that both isolated ADAMTS13 deficiency and DIC-associated ADAMTS13 deficiency were markers of severity in sepsis. Only one study dedicated to ADAMTS13 in 40 septic patients analyzed disease severity scores (LOD and SAP scores) but did not find any correlation with ADAMTS13 [23]: this discrepancy with our study may be related to the presence of only eight patients with septic shock and no cases of DIC [23].

Furthermore, in our study, there was also significant correlation between the number of hemostatic abnormalities and some outcome indicators that is, persistent shock at day 3. The need for dialysis, reflecting renal failure, was not significantly different between the three groups although the association of both DIC and ADAMTS13 deficiency <30% exhibited more renal failure than both of the other groups with a borderline significance (*P* = 0.05). These data are in agreement with other studies of the literature led in adult patients [20,21,24-26]: indeed, our group 3 is strongly comparable to the 109 patients with sepsis-induced DIC described by Ono *et al.*[20] who also found a higher rate of renal failure in patients whose ADAMTS13 activity was <20%. Also, Martin *et al.*[21] demonstrated that in 30 patients with severe sepsis, including 17 patients with septic shock and 4 patients with DIC, ADAMTS13 activity <30% was correlated with renal failure and as in our study, with hemodynamic shock. Other studies involving patients with severe sepsis/septic shock [24-26] also found a correlation between ADAMTS13 activity and organ dysfunction, in contrast to Kremer *et al.*[23].

Finally, our study demonstrates that DIC and ADAMTS13 deficiency <30% are independent markers of mortality in septic shock, particularly as the mortality rate is strongly dependent on the position of the ADAMTS13 level towards the median value (significantly higher mortality rate when ADAMTS13 is <30%) and ADAMTS13 deficiency appears to be a potential prognosis marker. Although prognostic association between ADMATS13 deficiency and outcome remains after adjustment for confounders, the diagnostic performance of this test remains modest. In addition, confirmation of our findings in a validation cohort is needed. To corroborate this observation, both ICU and mortality rates were not significantly different in group-2 (mainly including isolated ADAMTS13 deficiencies) and group-3 patients. In other words, in our cohort, the addition of DIC to ADAMTS13 deficiency did not significantly change the mortality rate. Again, these data are in agreement with most studies of the literature [20,21,24-26]. Interestingly, in our cohort, four patients exhibited ADAMTS13 activity <5%. A previous study in 109 patients with sepsis-induced DIC also found that 17 patients had severe ADAMTS13 functional deficiency [20], and also exhibited a TTP-like clinical presentation. The mechanism by which some septic patients reach a severe ADAMTS13 deficiency similar to TTP patients is very likely multifactorial and may also have an important impact on prognosis.

The association between functional partial deficiency of ADAMTS13 and clinical prognosis factors in sepsis raises the question of the relevance of this metalloprotease as a passive marker or, in contrast, as an actor in the pathophysiology of organ failure. First, normal ADAMTS13:Ag levels found in our cohort suggested that ADAMTS13 functional deficiency was mainly related to a qualitative defect and not to a quantitative defect secondary to a synthesis/secretion deficiency or to a degradation process mediated by sepsis-related enzymes [20,44-48]. Second, direct catalytic inhibition of ADAMTS13 by IL-6, a cytokine highly secreted in sepsis, has been reported *in vitro* using ULVWF strings secreted from human umbilical vein endothelial cells under flowing conditions [49]. Clinical studies focused on septic patients are controversial, showing either no correlation between ADAMTS13 activity and IL-6 levels [23] or in contrast, a strong inverse correlation between these parameters [21,24]. Interestingly, in the current study, we also found a strong inverse correlation between ADAMTS13 activity and IL-6 levels. Although this inverse correlation does not allow extrapolation of any causal relationship, this result suggests a potential role of IL-6 in the functional deficiency of ADAMTS13 observed in septic shock. Additional studies are needed to confirm these findings and to more clearly assess the role of IL-6 on ADAMTS13 activity. Also, in our study, the link between IL-6 levels and prognosis already reported by other authors [50] may be mediated by ADAMTS13 deficiency. In contrast, no relevant specific autoantibodies to ADAMTS13 were detected, making unlikely the involvement of an autoimmune process in sepsis-associated ADAMTS13 functional deficiency. Third, in all our patients, we found increased VWF:Ag levels and the presence of UL-VWF multimers in plasma. An overwhelming release of ULVWF multimers from activated endothelial cells may exhaust ADAMTS13 activity by a consumption mechanism [44,46,49-54]. In septic patients, the literature is controversial either supporting this consumption process [20,21,24,25] or not [23]. Further studies are needed to confirm prognostic impact of ADAMTS13 deficiency and to validate a cutoff for ADAMTS13 activity that may be used to assess outcomes, independently of other determinants of death.

Our study has several limitations. First, our study does not include any control group. Indeed, without this control group we do not know whether the current findings are specific to septic shock and whether the observed relationship (or lack thereof) is similar to the relationship in critically ill patients who do not have septic shock. In addition, the compared group had, as expected, different severity at study inclusion. However, the prognostic impact persisted after adjustment for patient severity, suggesting the prognostic association between ADAMTS13 deficiency and outcome to be independent from initial severity differences.

## Conclusion

Our study underlines that septic shock seems to be associated with partial functional deficiency of ADAMTS13, which mechanism is potentially related to IL-6-mediated inhibition but is independent of DIC. As a consequence, excessively released ULVWF multimers are more slowly cleared from the circulation and generate spontaneous formation of microvascular platelet thrombi responsible for multivisceral organ failure. Last but not least, our study emphasizes the role of ADAMTS13 as a prognosis factor in septic shock. These data are in agreement with the exciting concept of ADAMTS13 as a link between inflammation and thrombosis, which was recently developed thanks to animal models [55-58]. In addition, this study may bring new insights in the field of hemostasis inhibitor-based treatment in severe sepsis: indeed, although the efficiency of coagulation inhibitors is still controversial [59], downregulation of VWF-mediated thrombosis by exogenous ADAMTS13 brought by plasma infusions, or in the future by plasma-purified or recombinant ADAMTS13, may be an interesting new approach targeted on primary hemostasis.

## Key messages

• Septic shock seems to be associated with partial functional deficiency of ADAMTS13, which mechanism might be related to IL-6-mediated inhibition.

• ADAMTS13 is an independent prognostic factor during septic shock.

• No correlation was observed in this study between ADAMTS13 deficiency and DIC.

• ADAMTS13 deficiency may decrease clearance of excessively released ULVWF multimers, leading to spontaneous microvascular platelet thrombi formation and multiple organ failure.

## Abbreviations

ADAMTS13: A disintegrin and metalloprotease with thrombospondin type 1 repeats member 13; ADAMTS13:Ag: ADAMTS13 antigen; DIC: Disseminated intravascular coagulation; ELISA: Enzyme-linked immunosorbent assay; Ig: Immunoglobulin; IL-6: Plasma interleukin (IL)-6; ISTH: International Society for Thrombosis and Haemostasis; LOD: Logistic Organ Dysfonction score; MV: Mechanical ventilation; OR: Odds ratio; PaO2/FiO2: arterial partial pressure of oxygen/inspired oxygen fraction; PI: Prothrombin index; RRT: Renal replacement therapy; SAPS II: Simplified Acute Physiology Score 2; TMA: Thrombotic microangiopathy; TTP: Thrombotic thrombocytopenic purpura; ULVWF: Ultralarge von Willebrand Factor multimers; VWF: von Willebrand Factor; VWF:Ag: von Willebrand Factor antigen.

## Competing interests

The authors declare that they have no competing interests.

## Authors’ contributions

VP, EA, BS and AV conceived the study, participated in its design and coordination and helped to draft the manuscript. IC, EM, NA and MD participated in study design, patient recruitment and performed the statistical analysis. VP, PL, AMK, MW and AV carried out the biological testing and cytokine evaluation. All authors read and approved the final manuscript.
